# Editorial: New Trends in Early-Stage Lung Cancer Presenting as Ground-Glass Opacities: Clinical, Pathological and Molecular Aspects

**DOI:** 10.3389/fonc.2021.792252

**Published:** 2021-11-23

**Authors:** Kezhong Chen, Chen Chen

**Affiliations:** ^1^ Department of Thoracic Surgery, Peking University People’s Hospital, Beijing, China; ^2^ Department of Thoracic Surgery, The Second Xiangya Hospital of Central South University, Changsha, China

**Keywords:** lung cancer, ground-glass opacities, treatment, diagnosis, early-stage, multiple lesions, molecular change

Ground-Glass Opacity (GGO)-associated lung cancers are radiologically distinct clinical entities. Consensus or guidelines suggest that the clinical management decision on ground-glass nodules (GGN) should be based on the initial size, percentage of solid portion, and growth rate of GGNs, but are inconsistent in terms of the GGN cutoff size for surgical intervention and low-dose CT scan (LDCT) follow-up frequency (1–3). In addition, the unpredictable aggressive potential and highly heterogeneous characteristics of GGNs add another layer of complexity for GGN management. Thus, effectively treating GGNs remains challenging for clinicians who may have to rely on their individual experiences. To address this knowledge gap, we initiated this Research Topic to further explore the clinical manifestations, pathological features, and genetic changes that will assist in the diagnosis and decision-making of treatment of early-stage lung cancer presenting as GGOs. A number of interesting studies closely related to this field were included.

Previous studies have shown that most resected GGNs are histologically atypical adenomatous hyperplasia (AAH), adenocarcinoma *in situ* (AIS), minimally invasive adenocarcinoma (MIA), and lepidic predominant adenocarcinoma, which are considered to have a low risk for regional lymph node metastasis, vascular invasion, or pleural invasion. In the WHO 5^th^ lung cancer TNM clinical classification, AIS has been removed from preinvasive to precursor lesions, companied with AAH (4). Wang et al. reported that using a radiomics prediction model in patients with subcentimeter GGOs to distinguish AAH from early lung adenocarcinomas. The model turns to have the potentiality to improve preoperative prediction accuracy for AAH nodules and may help to avoid unnecessarily aggressive operations for patients with AAH.

Prospective multicenter validation with higher accuracy is needed for routine clinical application as surgeons are unwilling to defer surgery based on false-negative predictions that will delay the treatment of invasive lesions. GGNs with a solid component are more likely related to invasive adenocarcinoma with poor prognosis. Xi et al. and Tsutani et al. studies confirmed the solid size of GGNs has a decisive effect on prognosis and provided an accurate solid tumor size cutoff for high-risk patients. Qi et al. reported that in small-sized lung adenocarcinoma, consolidation tumor ratio (CTR) is the best sign for predicting lung cancer spread through air spaces (STAS).

While ensuring the treatment effect, reducing the extent of resection and improving the quality of life are both important issues in surgical research for early-stage lung cancer in recent years (5). Handa et al. reviewed the transition of treatment strategies for NSCLC with GGNs according to a series of clinical trials. The primary endpoint of clinical trial JCOG 0802 is the overall survival between segmentectomy and lobectomy for NSCLC patients with tumors less than 2cm. After a long time of patients’ enrollment, this trial finally presented results this year and showed a longer OS of segmentectomy for patients with GGN lesions, indicating further prompted limited resection may be an optimal choice (6). Wu et al. compared perioperative outcomes between precise and routine segmentectomy for GGNs and showed their advantages, respectively. Precise segmentectomy is a technique improvement, but whether it could bring oncological improvement is still unclear. In addition, the learning curve and technical barriers of precise segmentectomy may delay its widespread application in low-volume centers and for inexperienced thoracic surgeons.

Due to the widespread use of low-dose computed tomography and computer-aided detection/diagnosis systems (CADe/x), multiple pulmonary nodules have become an increasingly recognized phenomenon, especially GGNs frequently appearing as extremely multiple nodules (≥3), which presents a challenge for diagnosis and treatment. Two studies identified the phenomenon of different GGNs originating from the same clone in the same patient (7, 8). Those GGNs were all in close proximity which might result in dissemination along the airway. Whether these multiple GGNs sharing the same mutation affect the prognosis needs to be explored with longer follow-up. Wang et al. collected a large cohort of patients with large numbers of GGNs to investigate the clinical and pathologic features, surgical methods, and prognosis of these patients and found the proportion of malignant nodules did not increase significantly with the increasing number of nodules and no lymph node invasion was observed, which suggested that the number of nodules may not affect surgical strategy or prognosis, providing insights for the treatment strategy of such patients.

The indolent clinical course and superior survival of GGNs imply a unique underlying biology. However, the molecular characteristics of GGNs have not been systemically studied. Wei et al. systematically reviewed the molecular alterations in lung adenocarcinoma (LUAD) with GGNs and revealed the correlation between driver mutations and the radiological progression. Ouyang et al. reported that the occurrence of GGOs may be related to hereditary or genetic factors. A whole-exome sequencing of lung cancer performed as GGN revealed the key genetic mutational events that potentially maintain the relatively inert nature of GGN and its progression from GGN to aggressively advanced lung adenocarcinoma (8).

In addition to accumulating molecular alterations, cancer evolution is constantly shaped by the dynamic interaction between cancer cells and host factors, particularly immune surveillance. Zhang et al. found IL-6 expression status and NK cell levels of early lung adenocarcinoma as GGN is significantly reduced. Stimulation of IL-6 could activate NK cells, suggesting that the immune response in the tumor microenvironment might play a critical role in the development of GGOs. Wu et al. reported based on two cases, that synchronous GGNs may not be sensitive to anti-PD-1/PD-L1 based therapy. In this study, the proportion of CD8+T cells was lower in synchronous GGNs than in primary lung cancers, while tumor-associated macrophages showed significant enrichment in the tumor microenvironment of GGNs (9). A recently published study further performed multiomics analysis of a consecutive clinical cohort prospective observational cohort study to characterize pulmonary nodules with or without GGO component and found GGO-associated lung cancers with lower mutational burden, less active immune environment, and less ctDNA shedding, revealing that the intrinsic biological features may have contributed to the indolent clinical course of GGO-associated lung cancers. This supports the hypothesis that GGO may represent early carcinogenesis of a subset of LUADs when cancer cells and anti-tumor immune response are at equilibrium and provides mechanistic insights into the diagnosis and treatment of these radiologically distinct clinical entities (10). 

Because of the unique biological, psychological, and environmental characteristics of each patient, clinical management of GGNs should be customized to meet individual patients’ needs. As minimally invasive surgical technology improves, surgical resection of GGNs is becoming increasingly safe and less invasive, and the lung function and quality of life can also be maximally preserved. Surgical resection of GGNs removes the potential risk of malignant progression of the GGNs and relieves patients’ anxiety. Nevertheless, surgery is also associated with risks of postoperative complications. Thoracic surgeons should weigh the benefits and risks of surgical resection carefully before making a therapeutic decision. Careful consideration of the indication for surgery is crucial for the effective management of GGNs and to avoid overtreatment.

With several updates and novel findings, this Research Topic will provide new insight for a better understanding of the clinicopathological and molecular characteristics of GGOs. Surgeons and oncologists will broaden their knowledge and find new clues for the treatment of early-stage lung cancer presenting as single or multiple GGOs. Further investigations on the natural course of GGNs will undoubtedly improve our understanding of the unique biological characteristics of GGNs and thus provide clinical evidence for determining the optimal timing of surgical resection. In addition, studies on molecular and genetic mechanisms underlying the adverse progression of GGNs could reveal potential therapeutic targets to prevent or delay GGN progression.

## Author Contributions

KC and CC contributed to the conceptualization and writing the manuscript. All authors contributed to the article and approved the submitted version.

## Funding

KC is supported by the National Natural Science Foundation of China (No. 82072566) and Peking University People’s Hospital Research and Development Funds (RS2019-01). CC is supported by the Hunan Provincial Natural Science Foundation (No. 2020SK53419, No. 2019JJ50953 and No. 2021JJ30926), Hunan Provincial Key Area R&D Program NO. 2019SK2253, CSCO Cancer Research Foundation (CSCO-Y-young2019-034 and CSCO-2019Roche-073), and the Changsha Municipal Natural Science Foundation NO. kq2014246.

## Conflict of Interest

The authors declare that the research was conducted in the absence of any commercial or financial relationships that could be construed as a potential conflict of interest.

## Publisher’s Note

All claims expressed in this article are solely those of the authors and do not necessarily represent those of their affiliated organizations, or those of the publisher, the editors and the reviewers. Any product that may be evaluated in this article, or claim that may be made by its manufacturer, is not guaranteed or endorsed by the publisher.
